# Psychotropic medication non-adherence among patients with severe mental disorder attending at Bahir Dar Felege Hiwote Referral hospital, north west Ethiopia, 2017

**DOI:** 10.1186/s13104-019-4126-2

**Published:** 2019-02-26

**Authors:** Daniel Ayelegne Gebeyehu, Haregewoin Mulat, Lulu Bekana, Nega Tezera Asemamaw, Mequanent Kassa Birarra, Wubet Worku Takele, Dessie Abebaw Angaw

**Affiliations:** 1 School of Nursing, Department of Community Health Nursing, College of Medicine and Health Sciences, University of Gondar, Gondar, Ethiopia; 2 School of Medicine, Department of Psychiatry, College of Medicine and Health Sciences, University of Gondar, Gondar, Ethiopia; 3 Amanuel Mental Health Specialized Hospital, Addis Ababa, Ethiopia; 4 School of Nursing, Department of Pediatric and Child Health Nursing, College of Medicine and Health Sciences, University of Gondar, Gondar, Ethiopia; 5 School of Pharmacy, Department of Clinical Pharmacy, College of Medicine and Health Sciences, University of Gondar, Gondar, Ethiopia; 6 School of Public Health, Department of Biostatistics and Epidemiology, College of Medicine and Health Sciences, University of Gondar, Gondar, Ethiopia

**Keywords:** Medication non-adherence, Severe mental disorder, Ethiopia

## Abstract

**Objective:**

Medication non-adherence is a major public health problem that has been called an “invisible epidemic”. Globally, non-adherence rates among patients with severe mental illness ranged between 30 and 65%. It greatly increases the risk of illness exacerbation and hospitalizations. However, there is a paucity of studies examining treatment non-adherence and its associated factors among patients with severe mental disorder. Therefore, this study was aimed at determining the magnitude of medication non-adherence and associated factors among patients with severe mental disorder.

**Results:**

A total of 409 study subjects were participated making a response rate of 92%. The overall prevalence of medication non-adherence was found to be 55.2% (95%, CI; 49.9%, 60.2%). Study participants whose age group of (25–34) [AOR = 3.04 (95% CI 1.27, 7.29)], study subjects taking their medication twice per day [AOR = 4.60 (95% CI 2.25, 9.43)], no social support [AOR = 4.4 (95% CI 1.78, 11.08)] and no insight for their treatment [AOR = 5.88 (CI 2.08, 16.59)] were significantly associated with medication non-adherence. The result of this study showed that non-adherence among patients with severe mental disorder was found to be high. Psychiatry health care providers have to consider the frequency of medication become once per day, continual awareness creation among professionals and engaging significant others for good social support system and continual treatment alliance is strongly commended for adherence.

**Electronic supplementary material:**

The online version of this article (10.1186/s13104-019-4126-2) contains supplementary material, which is available to authorized users.

## Introduction

Currently, psychotropic medications are a vital component in the treatment of patients with severe mental disorders with the growing availability of a wide range of drugs to treat mental illnesses [1]. Based on the World Health Organization (WHO) definition of adherence, medication adherence is the extent to which a person’s behavior-taking medications, following the diet, and/or executing lifestyle changes, corresponds with agreed recommendations of a healthcare provider [2, 3].

Medication non-adherence has serious consequences for individuals as well as in the country, having psychiatric disorders often resulting in 3.7 time’s higher rates of relapse and exacerbation of psychotic symptoms, increased aggression and worse prognosis, more violent than adherent patients, higher hospitalization, and poorer community adjustment [4–7].

The problem of non-adherence is multi-factorial; some of which are, younger patients [8], being females, married, primary class education, unemployed [9], having perception being stigmatized, and the odds medication non-adherence is higher among substance, khat/‘chat’, alcohol and cigarette users [10].

In Ethiopia, there is a paucity of studies examining treatment non-adherence and its associated factors among patients with severe mental disorder in developing countries. Therefore, this study was aimed to assess the magnitude of medication non-adherence and to figure out the determinant factors. Furthermore, it would provide scientific evidence for local decision-makers, programmers and service planners to make an informed decision that would address the best interests of a patient with severe mental disorder leading to better health and quality of life.

## Main text

### Methods

#### Study design, study area and period

An institutional-based cross-sectional study was conducted from June 01, 2017 to June 30, 2017, G.C in Felege Hiwot Referral Hospital, Bahir Dar, North West Ethiopia, a capital city of Amhara Regional State of Ethiopia. It is located 563 km. far from Addis Ababa. According to the 2012 National population census, the city has a total of 549,429 populations [11]. From a report sheet, the average number of SMD patients who visit the outpatient department per month was 1100, among this 650, 200 and 250 are schizophrenic, bipolar and MDD, respectively.

#### Study population, sample size determination and sampling techniques

Patients with Schizophrenia, Bipolar and MDD who had at least two or more regular follow up at Bahir Dar Felege Hiwot Referral Hospital were included in the study. On the other hand, Individuals who were seriously ill, unable to respond to the interview and those who did not have family/caregiver were excluded.

The sample size was calculated using a single population proportion formula by considering the following statistical assumptions, the prevalence of medication non-adherence among adult psychiatric outpatient at Jimma was 41.2% [12], 95% confidence interval, and 5% degree of precision with a non-response rate of 10% which gives a total of 409 study participants.

The stratified sampling technique was employed. The proportional allocation was done basing the three disorders namely, Schizophrenia, Bipolar and MDD. Then after, the systematic random sampling technique was used to select study participants from each stratum.

#### Study variables, data collection procedures and tools

Psychotropic medication non-adherence was the dependent variable in which participants who were scored greater than or equal to 3 in 8-items of Morisky scale were considered as non-adherence [13, 14]. Social support was assessed using Oslo-3 Social Support Scale (OSS-3); interpretation was made as, a person scored 12–14 (good), 9–11 (moderate), and 3–8 (poor social support) [15]. Perceived internal stigma was considered when the patient who has scored one or more out of the 3-item instrument of screening perceived internal stigma [16].

Medication attitude inventory (DAI), a 10-item true/false scale; was employed to assess beliefs about treatment. A correct answer scored as + 1, and an incorrect answer scored as − 1. The final DAI score is the sum of the pluses and minuses. A positive total score indicates a positive attitude towards medication, and a negative total score, a negative attitude [14].

Moreover, Insight was measured by using three item questions which were adopted from a study conducted in Nigeria, Africa. After checking its validity by taking a pre-test and modified it into our context. The interpretation was made as 1 was coded as yes and zero for no. The total score ranged between 0 and 3. After summation of the scores, the interpretation was made based on; a score of zero is regarded as “no insight”, a score of 1–2 is “partial insight”, and a total score of 3 as “full insight” [17].

#### Data quality assurance, data processing, and analysis

The quality of data was assured by providing training for data collectors and supervisors, pretest and by translating the questionnaire into local language, Amharic. Data were checked for completeness and its consistency. After appropriate coding, the collected data has been entered into epiinfo version 7 and it was exported to SPSS version 23 for analysis. The Binary logistic regression model was fitted to control the effect of confounders; variables which had a *P* value of less than 0.2 in the bi-variable model were entered to the multi-variable regression model. Finally, variables having a P-value of < 0.05 at 95% CI in the multivariable binary logistic regression were considered as statistically significant.

### Results

#### Socio-demographic characteristics

About half (50.7%) of the study participants were male. The mean (± SD) age of participants was 33 (± 12.16) years with a range of 18 and 76 years. The majority (40.3%) of the respondents were in the age category of 25–34 years. Among the total participants, about (81.4%) were orthodox (49.3%) single, and (35.5) can’t read and write (Table 1).

**Table 1:** Socio-demographic characteristics of patient with severe mental disorder attending at Bahir Dar Felege Hiwot hospital, outpatient psychiatric department, April 2017

Variable	Frequency	Percentage
*Sex*		
Male	191	50.7
Female	186	49.3
*Age*		
18–24	84	22.3
25–34	152	40.3
35–44	80	21.2
≥ 45	61	16.2
*Mean age 33 (sd *±* 12.16)*		
*Religion*		
Orthodox	307	81.4
Muslim	64	17.0
Protestant	6	1.6
*Marital status*		
Single	186	49.3
Married	129	34.2
Separated	12	3.2
Divorced	37	9.8
Windowed	13	3.4
*Educational status*		
Can’t read and write	134	35.5
*Grade*		
1–8	84	22.3
9–12	82	21.8
Diploma and above	77	20.4

#### Treatment-related and illness factors

**Fig. 1:**
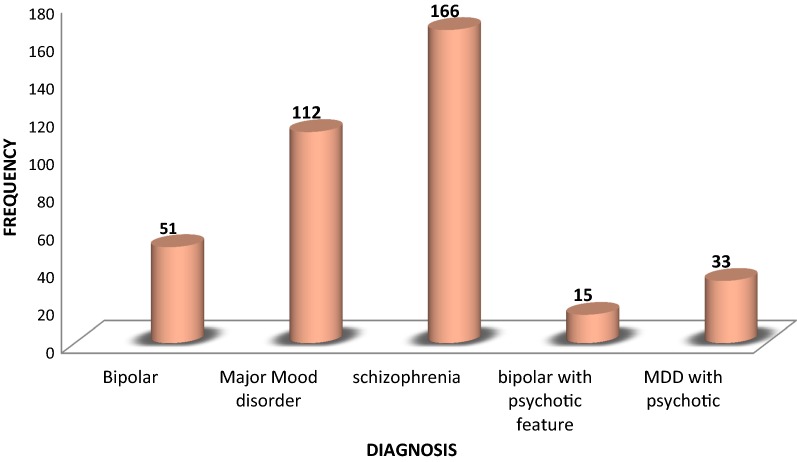
Diagnosis among patients with severe mental disorder attending at Bahir Dar Felege Hiwot hospital, outpatient psychiatric department, April 2017

Related to clinical characteristics of the study participants, the greatest (44.0%) numbers of participants were suffering from schizophrenia (Fig. 1). The vast majority 274 (72.7%) of participants were taking their medication ones per day, whereas, 99 (23.9%), and 3 (0.8%) were two times and three times per day, respectively. Nearly two-thirds of participants 239 (63.4%) were greater than 12 months on treatment follow up, the rest 68 (18%) and 70 (18.6%) had less than 6 and 6–12 months, respectively.

#### Types of currently used medication and side effects experienced by the study participants

In the types of medication currently used among patients with severe mental disorder, majority of them were taking typical antipsychotic and antidepressant 87 (23.1%) (Additional file 1).

More than half (58.4%) of the study participants have not experienced side effects to the medications. The most frequently occurred side effects were tiredness; excessive sleep and weight gain 25.5% (Additional file 2).

#### Healthcare and patient-related factors

Concerning the consequences of non-adherence, about 232 (61.5%) knew the consequences of medication non adherence. More than half 213 (56.5%) of participants were not received health education about their treatment. Among the participants 167 (44.3%) reported that the expense of medication is cheap, whereas, 81 (21.5) reported that expensive and the rest 129 (34.2%) to get their medication freely.

Regarding substance use during the treatment period, 80.1% of the study participants had experiences of substance use. Among users, 20.3%, 19.4% and 9.6% were used alcohol, chat and cigarette. However, 12.2%, 15.5% and 4.0% of the participants were multi-substance users that had chat and cigarette, chat and alcohol and all the three, respectively. More than half (57.6%) of participants experienced skipping of their medication (Additional file 3).

#### Patient/family related factors

Nearly three out of ten (25.7%) participants had perceived stigma. Of 45.9% had good social support and relation and (70%) had a positive attitude towards their medication. Concerning insight, more than half (55.4%) of the participants had good insight towards their illness and treatment (Additional file 4).

#### Prevalence of medication non-adherence

The overall prevalence of medication non-adherence was found to be 55.2% (95%, CI; 49.9%, 60.2%).

#### Factors associated with medication non-adherence

The odds of developing medication non-adherence among the age group of (25–34) was 3.04 (AOR = 3.04 (95% CI 1.27, 7.29) times higher as compared to participants age older than 45 years.

The likelihood of having medication non-adherence among patients taking their medication twice per day was 4.6 (AOR = 4.60 (95% CI 2.25, 9.43) times higher as compared to those taking once per day.

The likelihood of medication non-adherence among patients who had no social support were about 4.44 (AOR = 4.4 (95% CI 1.78, 11.08) times higher as compared to those participants who had good social support. The odds of medication non-adherence among patients who had no insight for their treatment were about six (AOR = 5.88 (CI 2.08, 16.59) folds higher as compared to their counterparts (Table 2).

**Table 2:** Multivariable factors associated with medication non-adherence among patient with severe mental disorder attending at Bahir Dar Felege Hiwot hospital (n = 377)

Explanatory variables	Med. non adherence		COR (95% CI)	AOR (95% CI)
	Yes	No		
*Age*				
18–24	44	40	1.29 (0.67, 2.51)	1.34 (0.53, 3.44)
25–34	96	56	2.02 (1.10, 3.69)	3.04 (1.27, 7.29)
35–44	40	40	1.18 (0.60, 2.29)	2.45 (0.93, 6.59)
≥ 45	28	33	1	1
*Frequency of medication*				
Once per day	70	33	1	1
Two times per day’	138	136	2.09 (1.29, 3.37)	4.60 (2.25, 9.43)
*Use of active substance*				
Yes	155	145	0.48 (0.28, 0.82)	
No	53	24	1	
*Social support*				
Poor	128	80	3.54 (1.92, 6.51)	4.44 (1.78, 11.08)
Intermediate	61	47	2.87 (1.48, 5.56)	3.39 (1.28, 9.04)
Good	19	42	1	1
*Attitude towards medication*				
Positive	132	132	1	
Negative	76	32	0.42 (0.31, 0.77)	
*Insight*				
No	55	8	12.28 (5.55, 27.16)	5.88 (2.08, 16.59)
Partial	78	27	5.16 (3.07, 8.69)	2.88 (1.38, 6.01)
Good	75	134	1	1

### Discussion

Typically, patients with a psychiatric disorder, almost in all have a major problem following a medication regimen [18, 19]. So, this study examined the magnitude of psychotropic medications non-adherence and its associated factors among patients with severe mental disorder.

The current study found that 55.2% of patients with a severe mental disorder were non-adhere with their medication. This finding was in line with global non-adherence rates among patients with severe mental illness between 30 and 65% [20, 21]. This finding was also consistent with a study conducted in Nigeria 54.2% [22], and 55.7% [17]. However, lower than Pakistan’s study (64.75%) [8]. This variation could be the differences in socio-demography of the study population, selection criteria for the study participants and the lower sample size were taken in Pakistan, such as a convenient, non-probability sample of one hundred and thirty-five follow-up patients were assessed [8]. The non-adherence rate in the present study was found to be further an up than a report from in Jimma, Ethiopia 41.2% [12], and India 26% [23]. This discrepancy could result from the methodological differences in the studies, such as the tools used for the assessment of adherence. In Jimma, i.e. Morisky 4 item scale was used and health care services.

This study comes up with psychotropic medication non-adherence was significantly associated with the age group of 25–34 years. This finding is in agreement with studies conducted in Qatar [24], India [25] and USA [26]. This might be being younger is prone a patient not to adhere the medication by considering the negative attitudes towards the medication as well the illness. It is also supported by; that individuals who have a negative perception towards their medication perceive themselves to have a capacity to control or manage their problem on their own [27].

This study was revealed that participants who were taking their medication twice daily were significantly associated with non-adherence. It was also in line with the study conducted in Ethiopia [12], Nigeria [22], India [25] and USA [26]. This might be due to forgetting to take their medication, fear of side effects and the discomfort arising from the high ‘pill burden’ fear of pill overload [17].

Regarding social support, participants who had no social support were significantly associated with non-adherence. This finding was supported by the study reported in Ethiopia [28], Nigeria [29], and USA [30, 31]. The possible explanation for this might be having social support as a cue to alarm them to take their medication on time, by minimizing fear that was related to medication side effects and by educating them the consequence of medication non-adherence.

Patients who had no insight into their disorder and treatment were significantly associated with medication non-adherence. This result is also similar to the study reported in Nigeria [28] India [25, 32, 33], USA [34] and Germany [35]. The possible explanation for this might be having or presence of insight towards the disease and their treatment plays an important role in medication adherence.

### Conclusion

The result of this study showed that non-adherence among patients with severe mental disorder was found to be high. Psychiatry health care providers have to consider the frequency of medication become once per day, continual awareness creation among professionals and engaging significant others for good social support system and continual treatment alliance is strongly commended for adherence.

#### Limitations of the study

The limitation of this study might be affected by recall bias. This might distort the actual outcome.

## Additional files

**Additional file 1.** Types of medication currently used among patients with severe mental disorder attending at Bahirdar Felege Hiwot hospital, outpatient psychiatric department, April 2017.

**Additional file 2.** Side effects profile self-reported by patient with severe mental disorder attending at Bahirdar Felege Hiwot hospital, outpatient psychiatric department, April 2017.

**Additional file 3.** Reason for psychotropic medication non-adherence among patients with severe mental disorder attending at Bahirdar Felege Hiwot hospital, outpatient psychiatric department, April 2017.

**Additional file 4.** Patient/family related factors of patient with severe mental disorder attending at Bahirdar Felege Hiwot hospital, outpatient psychiatric department, April 2017.

## Abbreviations

AMSH: Amanuel Mental Specialized Hospital
BPD: bipolar disorder
CI: confidence interval
COR: crude odds ratio
OPD: out patient department
OR: odds ratio
UOG: University of Gondar
WHO: World Health Organization
