# Supplementary material for: Psychotropic medication non-adherence among patients with severe mental disorder attending at Bahir Dar Felege Hiwote Referral hospital, north west Ethiopia, 2017
Source: BMC Res Notes. 2019 Feb 26;12:102. doi: 10.1186/s13104-019-4126-2 (PMC6390330; doi:10.1186/s13104-019-4126-2)
Supplement: Supplementary file 1 — Additional file 1. Types of medication currently used among patients with severe mental disorder attending at Bahirdar Felege Hiwot hospital, outpatient psychiatric department, April 2017. [file 13104_2019_4126_MOESM1_ESM.docx]

Figure S1: Types of medication currently used among patients with severe mental disorder attending at Bahirdar Felege Hiwot hospital, outpatient psychiatric department, April 2017
