# Supplementary material for: Psychotropic medication non-adherence among patients with severe mental disorder attending at Bahir Dar Felege Hiwote Referral hospital, north west Ethiopia, 2017
Source: BMC Res Notes. 2019 Feb 26;12:102. doi: 10.1186/s13104-019-4126-2 (PMC6390330; doi:10.1186/s13104-019-4126-2)
Supplement: Supplementary file 2 — Additional file 2. Side effects profile self-reported by patient with severe mental disorder attending at Bahirdar Felege Hiwot hospital, outpatient psychiatric department, April 2017. [file 13104_2019_4126_MOESM2_ESM.docx]

Table S1: side effects profile self-reported by patient with severe mental disorder attending at Bahirdar Felege Hiwot hospital, outpatient psychiatric department, April 2017

| Variable | Frequency | Percentage |
| --- | --- | --- |
| EPS | 12 | 7.6 |
| dry mouth | 10 | 6.3 |
| Dizziness | 4 | 2.5 |
| tiredness and excessive sleep | 37 | 23.5 |
| Sleeplessness | 8 | 5.4 |
| weight gain | 12 | 7.6 |
| tiredness, excessive sleep, and weight gain | 40 | 25.5 |
| EPS, tiredness and excessive sleep | 30 | 19.1 |
| dry mouth and constipation | 4 | 2.5 |
