# Supplementary material for: Psychotropic medication non-adherence among patients with severe mental disorder attending at Bahir Dar Felege Hiwote Referral hospital, north west Ethiopia, 2017
Source: BMC Res Notes. 2019 Feb 26;12:102. doi: 10.1186/s13104-019-4126-2 (PMC6390330; doi:10.1186/s13104-019-4126-2)
Supplement: Supplementary file 3 — Additional file 3. Reason for psychotropic medication non-adherence among patients with severe mental disorder attending at Bahirdar Felege Hiwot hospital, outpatient psychiatric department, April 2017. [file 13104_2019_4126_MOESM3_ESM.docx]

Figure S2: Reason for psychotropic medication non-adherence among patients with severe mental disorder attending at Bahirdar Felege Hiwot hospital, outpatient psychiatric department, April 2017
