# Supplementary material for: Psychotropic medication non-adherence among patients with severe mental disorder attending at Bahir Dar Felege Hiwote Referral hospital, north west Ethiopia, 2017
Source: BMC Res Notes. 2019 Feb 26;12:102. doi: 10.1186/s13104-019-4126-2 (PMC6390330; doi:10.1186/s13104-019-4126-2)
Supplement: Supplementary file 4 — Additional file 4. Patient/family related factors of patient with severe mental disorder attending at Bahirdar Felege Hiwot hospital, outpatient psychiatric department, April 2017. [file 13104_2019_4126_MOESM4_ESM.docx]

Table S2: patient/ family related factors of patient with severe mental disorder attending at BahirdarFelegeHiwot hospital, outpatient psychiatric department, April 2017

| Variable | Frequency | Percentage |
| --- | --- | --- |
| Perceived stigma  Yes  No | 152  225 | 40.3  59.7 |
| Social support  poor  intermediate  good | 208  108  61 | 55.2  28.6  16.2 |
| Attitude towards medication  Positive  Negative | 325  52 | 86.2  13.8 |
| Insight  Good  Partial  No | 209  105  63 | 55.4  27.9  16.7 |
